# Fatigue Evaluation through Machine Learning and a Global Fatigue Descriptor

**DOI:** 10.1155/2020/6484129

**Published:** 2020-01-06

**Authors:** G. Ramos, J. R. Vaz, G. V. Mendonça, P. Pezarat-Correia, J. Rodrigues, M. Alfaras, H. Gamboa

**Affiliations:** ^1^PLUX Wireless Biosignals S.A, Avenida 5 Outubro 70, 1050-59 Lisbon, Portugal; ^2^Department of Biomechanics and Center for Research in Human Movement Variability, University of Nebraska at Omaha, Omaha, NE, USA; ^3^Universidade Europeia, Laureate International Universities, Lisbon, Portugal; ^4^Neuromuscular Research Lab, CIPER, Faculty of Human Kinetics, University of Lisbon, Lisbon, Portugal; ^5^Laboratory for Instrumentation, Biomedical Engineering and Radiation Physics (LIBPhys-UNL), Faculty of Sciences and Technology of NOVA University of Lisbon, Caparica, Portugal; ^6^Universitat Jaume I, Castelló de la Plana, Spain; ^7^Department of Physics, Faculty of Sciences and Technology of NOVA University of Lisbon, Caparica, Portugal

## Abstract

Research in physiology and sports science has shown that fatigue, a complex psychophysiological phenomenon, has a relevant impact in performance and in the correct functioning of our motricity system, potentially being a cause of damage to the human organism. Fatigue can be seen as a subjective or objective phenomenon. Subjective fatigue corresponds to a mental and cognitive event, while fatigue referred as objective is a physical phenomenon. Despite the fact that subjective fatigue is often undervalued, only a physically and mentally healthy athlete is able to achieve top performance in a discipline. Therefore, we argue that physical training programs should address the preventive assessment of both subjective and objective fatigue mechanisms in order to minimize the risk of injuries. In this context, our paper presents a machine-learning system capable of extracting individual fatigue descriptors (IFDs) from electromyographic (EMG) and heart rate variability (HRV) measurements. Our novel approach, using two types of biosignals so that a global (mental and physical) fatigue assessment is taken into account, reflects the onset of fatigue by implementing a combination of a dimensionless (0-1) global fatigue descriptor (GFD) and a support vector machine (SVM) classifier. The system, based on 9 main combined features, achieves fatigue regime classification performances of 0.82 ± 0.24, ensuring a successful preventive assessment when dangerous fatigue levels are reached. Training data were acquired in a constant work rate test (executed by 14 subjects using a cycloergometry device), where the variable under study (fatigue) gradually increased until the volunteer reached an objective exhaustion state.

## 1. Introduction

Fatigue occurs frequently in several functional tasks performed on a daily basis [1]. Fatigue can be classified as objective or subjective, taking into consideration its nature [2].

Objective fatigue is a physical phenomenon, not only on its origins but also in the effects that it produces (generating a decrease in the capability to exert mechanical work), in opposition to subjective fatigue, which can be directly caused by intense or stressful mental tasks and, indirectly, by physical activities whose consequences, as De Luca [3] affirmed, are *“…characterized by a decline of alertness, mental concentration, motivation, and other psychological factors…”*.

Taking into consideration the relevance of sports in the modern society and the fact that it is also a valuable example of how physical activities may force the human body to reach its limits, the brave effort of athletes to reach their maximum performance was a great inspiration for this study.

However, in spite of the great preponderance of the physical component, athletes can only reach their full potential when mentally and physically healthy, reason why studying both subjective and objective fatigue becomes interesting to achieve a global evaluation of the consequences of fatigue and eventually adjust the training programs.

Through the previous descriptions, these two types of fatigue (objective and subjective) gradually rises due to an intense physical or mental activity, causing the body to reach its limit breaking the homeostasis state, due to the deficit between the metabolic energy production/consumption and the accumulation of metabolic waste at cellular level, conditioning the normal functioning of the organic system. The perturbation of the homeostasis state becomes a potential/transient cause of damage to the organism or, in more drastic situations, the effect of fatigue may be prolonged leading to overwork, chronic fatigue syndrome, overtraining syndrome, and immunity dysfunctions, as stated by Wan et al. [4].

In the specific case of objective fatigue, it reflects the inability of the organism to maintain homeostasis [4, 5] and may result from the physiological consequences of exercise (e.g., accumulation of metabolites, such as lactic acid [4], within the exercising muscle). Objective fatigue can be subdivided accordingly to its physiological origin in central fatigue, which reflects changes at the neuronal level that affect nerve conduction to the exercising muscle and in peripheral fatigue that is typically associated with changes in sarcoplasmic ionic concentrations [4, 6]. Regardless of its origin, fatigue is consistently accompanied by decreased physical performance [4, 7] caused by the imbalance between the rate of energy production/consumption and also by a deficit in the recycling of metabolic waste [8]. Objective fatigue (muscle level) may be more or less transient, depending on the characteristics of the exercise stimulus (i.e., intensity, duration, and volume) [9].

Regarding subjective fatigue, its effects were well defined since early studies, namely, drowsiness, inability to concentrate, and physical discomfort [10]; so, a possible definition that aggregates these three types of effects will establish subjective fatigue as a physical incapacity caused by psychological factors, conducting to subjective feelings of exhaustion influenced by loss of motivation or concentration [7, 11, 12].

In spite of these valuable definitions, for evaluating fatigue in a precise and reliable way, more concrete and unbiased approaches are needed, something achievable by the conjugation of machine and human powers.

Nowadays, artificial decision support systems extend the capabilities of the natural ones. With the help of machines, solving complex challenges delineated by the human brain is now much more reliable and time inexpensive. As knowledge benefits from the processing power of machines, incredible discoveries may arise, with great impact to the daily life of populations.

Following this line of thought, the current research article tries to explore fatigue, both as an objective and subjective physiological phenomenon (research variable) through the powerful capabilities of computational systems, using a conjugation of electromyographic (EMG) and electrocardiographic (ECG) signal processing methodologies together with the training of an exploratory machine-learning system.

Since past research has shown that muscle injuries are more frequently associated with prolonged forms of muscle fatigue [13], there is clearly a need for a monitoring system to identify the onset of fatigue, both in objective and subjective terms, using an indirect and noninvasive approach.

The proposed detection model included both electromyographic signals and heart rate variability (HRV) parameters because fatigue develops at the muscle level whenever the rate of oxygen delivery and utilization become imbalanced [5]. This global monitorization of the fatigue phenomenon (objective and subjective types) may have a major impact on the training programs of high-performance athletes, taking into consideration that, as previously mentioned, the maximum performance can only be achieved when subjects are physically and mentally healthy [14–16].

Fatigue can be evaluated through different methodologies (beyond EMG and HRV), distinguishable by the nature of studied events. As a practical example, muscle contraction is triggered by an (1) electrical impulse that generates (2) chemical changes at cellular level during its propagation in order to produce (3) a mechanical event, i.e., the muscular contraction. The previous three points are an illustrative demonstration of how fatigue acquisition is dependent on (1) electrical, (2) chemical, and (3) mechanical phenomena.

Interesting studies were also conducted regarding the mechanical and chemical nature of fatigue, namely, the research studies of Faller et al. [17] and Kimura et al. [18] (mechanical perspective) using accelerometric and acoustic sensors, respectively. It was concluded that, typically, the amplitude of the mechanical signal decreases as fatigue was being acquired.

From the chemical point of view, the use of near-infrared spectroscopy arises as a noninvasive methodology to measure relative changes in the oxygen levels at the muscular level. In spite of the concentrations of oxyhemoglobin and deoxyhemoglobin not evolving in a linear way, a specific 4-stage pattern was found by Taelman et al. [19].

The choice of ECG/HRV and EMG data to study fatigue is framed on the set of studies focused on the “electrical” nature of fatigue, providing a way to understand this phenomenon from its source and through multiple fatigue categories (objective and subjective types).

However, the current study is not restricted to a quantitative point of view, which may not be appropriate, taking into consideration that a specific value of consensual indicators of fatigue may flag the existence of fatigue for a segment of subjects, while for others it is innocuous, i.e., fatigue-related values may vary accordingly to the subject and experimental conditions under analysis. Due to this difficulty, it was decided to explore fatigue phenomenon from a qualitative and more universal point of view, through the analysis of how certain parameters evolve in time, instead of focusing on its absolute values. Since median frequency represents a valid parameter for monitoring muscle fatigue [5], the median frequency of the EMG signal power spectrum was one of the parameters where trends were searched, together with other documented wavelet parameters [20].

We extended this trend evaluation to linear and nonlinear HRV parameters (e.g., low- and high-frequency power as well as short- and long-term dispersion derived from the Poincaré analysis, respectively) extracted from the cardiac epochs (RR) obtained on a cardiopulmonary cycloergometry trial above the second ventilatory threshold (VT_2_). After identifying the most significant EMG and HRV parameters to evaluate fatigue, we aim to objectively classify the physiological impact of fatigue in the performance of athletes. A successful achievement of this purpose may ensure a way to study possible adaptations on training programs responsible for simultaneously improving athletes' physical indexes and minimizing the long-term impact of fatigue, avoiding severe injuries.

As previously mentioned, a preventive approach that ensures the monitorization of fatigue-related patterns can be followed by developing a computational system capable of extracting individual fatigue descriptors (IFD) from EMG and HRV signals, obtained during a cycloergometer exercise performed above the second ventilatory threshold.

EMG parameters may ensure a local/muscular monitorization of this phenomenon, while HRV indexes are quite relevant for a generalized/mental evaluation of the fatigue state [4, 16, 21]. The implemented system reflects the gradual onset of fatigue by incorporating a global fatigue descriptor (GFD) and a support vector machine (SVM) classifier, both supported by the evolution time series of IFD's (such as EMG median frequency) generated along the cycling exercise trial.

## 2. Materials and Methods

It should be taken into account that the following experimental environment/protocol replicated some conditions and procedures described on a past research work. One of the authors gave his contribution in the design of the study and also in the analysis of the results [64].

### 2.1. Participants

Fourteen healthy, young men (age: 24.5±3.6 years; body mass index: 23.7±1.7 kg·m^−2^) were included in this study. Participants were recruited from a local university and community population through direct invitation or flyers. From this population, a primary group of study was chosen and all volunteers were accustomed to cardiopulmonary exercise.

All participants were nonsmokers and normotensive (systolic and diastolic blood pressure values repeatedly < 120/80 mmHg) [22].

Participants were all nonoverweight and free of any known cardiovascular or metabolic disease, as assessed by medical history. None of the participants were currently using prescription or taking any medications or nutritional supplements. Taking into consideration one of the previously mentioned inclusion criteria, participants were already accustomed to cardiopulmonary exercise testing using a cycloergometer. For this reason, there were no familiarization sessions for this specific group of participants.

Each participant was requested to avoid heavy exercise for at least 24 h before testing and to have nothing to eat from midnight until the testing session on the subsequent morning (fasting conditions). Participants were also asked to refrain from caffeine ingestion and to empty their bladders before testing.

Participants were fully informed of the purposes, risks, and discomfort associated with the experiment before providing written informed consent. Informed consent was obtained from all individual participants included in the study. This study was carried out with the approval from the University's Institutional Review Board and in accordance with the Declaration of Helsinki.

### 2.2. Protocol

Participants were evaluated over the course of 2 visits on separate days (within a 7-day period). Testing was separated by at least 48 h and, to minimize the effects of circadian and other similarly induced variations in performance, was performed at approximately the same time of the day (between 07.00 and 11.00 a.m.).

During the first visit, standing height and weight measurements were taken with participants wearing light-weight clothing and no shoes. Height was obtained using a stadiometer with measures obtained to the nearest 0.5 cm. Weight was measured by using a digital scale (BG 42; Beurer GmbH, Söflinger, Germany). Body mass index was calculated by dividing the participants' mass in kilograms by the square of their height in meters. Subsequently, participants performed a treadmill-graded exercise test to determine their VT_2_ and peak oxygen uptake (VO_2peak_).

On the second visit, each participant performed one bout of cycloergometer exercise above the VT_2_ to volitional exhaustion. All tests were performed on the same cycloergometer (Monark Ergomedic 839E, Varberg, Sweden). Expired gas measurements were made using a portable mixing chamber (Metamax® I, Cortex, Leipzig, Germany), which was calibrated before each test with a known volume and with known gas concentrations. Testing was carried out in the laboratory with an environmental temperature between 21–24°C and a relative humidity between 44–56%.

In synthesis, *Day 1* dedicated to the execution of a “Graded Exercise Test” was focused on the assessment of participants' work rate (WR) near the second ventilatory threshold (VT_2_), through an incremental power test, where the cardiorespiratory fitness level was inferred.

As stated by Mourot et al. [23], the second ventilatory threshold is *“…the point where high-intensity exercise can no longer be sustained…”*. This parameter can be determined through the collection and monitorization of exhaled air, focusing the analysis on changes in oxygen and carbon dioxide concentrations. With this continuous monitorization, the determination of VE/VCO_2_ and VE/VO_2_ ventilatory equivalent parameters can be achieved and consequently the VT_2_.

On *Day 2*, a “Constant Work Rate Test” was conducted, taking into consideration the WR_VT_2+15%__ power, producing for each participant a set of physiological signals (EMG and HR analysis), where fatigue pattern search took place through digital processing.

#### 2.2.1. Graded Exercise Test (Day 1)

Graded exercise testing was performed using an incremental cycloergometer ramp protocol. Following a 3 min warm-up period at 60 W, work rate was increased by 15 W·min^−1^ until the participant was unable to continue. The participants cycled at a self-selected pedal rate between 60–90 rev·min^−1^, being constantly encouraged by the researcher to proceed the exercise in the desired rhythm.

The test was stopped when the pedaling rate could no longer be maintained. VO_2peak_ was defined as the highest VO_2_ attained in a 20 s average. Participants were considered to have reached VO_2peak_ if at least three of the following four criteria were met: (1) plateau (increase of no more than 150 mL·min^−1^) in VO_2_ with an increase in workload, (2) respiratory exchange ratio greater than 1.1, (3) peak heart rate within 10 bpm of the age predicted maximum, and (4) visible exhaustion [24].

All participants met at least three of these criteria. The peak heart rate was identified as the highest value recorded during each test. Additionally, using the time course of the relationship between the ventilatory equivalents for oxygen (Ve/VO_2_) and carbon dioxide (Ve/VCO_2_), the VT_2_ for each participant was determined by two independent investigators [25].

The VT_2_ was defined as the minimal work rate at which the Ve/VO_2_ as well as the Ve/VCO_2_ exhibited a systematic increase. The VT_2_ was expressed in absolute (W) intensity and used for defining the work rate to be used by each participant in the trial of Day 2 (WR_VT_2+15%__=1.15 × *P*_VT_2__). Participants included in this study exhibited a VO_2peak_ of 52.5±5.7 mL kg^−1^·min^−1^ and work rate at VT_2+15%_ =204.5±33.7 W.

#### 2.2.2. Constant Work Rate Test (Day 2)

On the second day, cycloergometry was performed at a constant work rate (WR_VT_2+15%__) and with a periodical encouragement by the researcher until volunteer reaches a volitional exhaustion.

During exercise, the cycling cadence was kept constant after setting the most appropriate value in response to a brief warm-up phase that preceded the test.

The participants were instructed to synchronize their right lower limb to an auditory metronome provided through speakers. Specifically, warm up was divided in 2 sets of 3 min, separated by 1 min rest, during which the participants exercised at their preferred cycling cadence. Then, the mean cadence recorded during the 6 min of warm up was used as the reference cadence for performing the constant work rate test.

The EMG signals from five lower limb muscles (*rectus femoris, vastus lateralis, vastus medialis, semitendinosus*, and *biceps femoris*) were acquired during exercise.

In the current stage of the research, as it will be explained with more detail, only the *rectus femoris* and *vastus medialis* data were taken into consideration during the formal definition of the proposed solutions (GFD and SVM) and methodologies (fatigue trend identification).

The acquisition was carried out using Ag/AgCl disposable electrodes and a signal acquisition system (biosignalsplux, PLUX S.A., Lisbon, Portugal) of PLUX Wireless Biosignals [26], following the recommendations of SENIAM (surface EMG for noninvasive assessment of muscles) [27]. The acquisition system followed the directives of the International Society of Electrophysiology and Kinesiology (acquisition at a sampling frequency of 1000 Hz, filtering using a band-pass filter between 10 and 500 Hz and common mode rejection ratio of 110 dB). In parallel, we also obtained HRV data using a Polar RS 800 G3 heart rate monitor (Polar R-R recorder, Polar Electro, Kempele, Finland).

Among the 14 participants of the study population, HRV data of 3 of them, collected from the constant power test of “Day 2,” were compromised due to loss of heart rate signal, so all HRV analysis and subsequent conclusions took into consideration a smaller population. Thus, when a combined analysis of EMG and HRV parameters was needed, it only included the participants with valid EMG and HR data, which were 11.

### 2.3. Signal Processing and Analysis

Besides data collection, our study was divided into three stages: (a) *Processing Stage*, (b) *Analysis Stage*, and (c) *Proposed Solutions*.

In the *Processing Stage*, a list with commonly extracted parameters from an EMG signal and HRV data was identified together with the computational methodology used to explore the existence of trends in these parameters over time.

The subsequent *Analysis Stage* covers the identification of which EMG and HRV parameters evolve over time in a characteristic trajectory with the progression of fatigue. First, we generated the evolution series of each parameter contained in the EMG and HRV *Processing Stage* preliminary list using a sliding window mechanism. We implemented an algorithm similar to Thongpanja et al. [28] and subsequently identified trends in the series using a linear regression model [29].

#### 2.3.1. Processing Stage - Features and Sliding Window Mechanism

From the EMG signal and HRV data, specific events had been selected (muscular activation periods and *R* peak positions, respectively).

For EMG, we followed the approach proposed by Pimentel et al. [30], which uses the Teager–Kaiser Energy Operator (TKEO).

For HRV, the *R* wave peaks, which we used to build the tachogram (time series with the duration of each RR interval along the acquisition – RR_tachogram_) were identified through the algorithm proposed by Pan and Tompkins [31].

After this process, a sliding window model was used for extracting samples of a set of EMG and HRV parameters over time, in order to explore trends. The window of dimension WS_*z*_ slides over each sample of the time series, taking into consideration a defined overlapping factor, dependent of the chosen time-step TS_*y*_, between consecutive windows [32].  Table 1 lists the parameters that were extracted from the EMG signal and HRV data.

A detailed description of HRV parameters can be found in Acharya et al. study [33] and Task Force of The European Society of Cardiology and the North American Society of Pacing and Electrophysiology [34]. A more exhaustive explanation for the EMG parameters, both from the temporal and the frequency domains, can also be found in Cifrek et al. [5].

Time-frequency information can be gathered through the wavelet transform, which shares some principles with the conventional Fourier analysis, namely, the decomposition of a signal into multiple elementary frequency components, mathematically ensured by a sequence of inner products between the signal and the “base” function (measuring their similarity).

However, while the “basis” of the Fourier analysis are sinusoidal functions, the wavelet transform provides more freedom to the researcher, taking into consideration that the elementary decomposition function (wavelet) can be chosen according to the characteristics of the signal to be decomposed.

Additionally, in the wavelet domain, some temporal information is preserved, in contrast with the Fourier domain being the Morlet mother wavelet (used on the current study) explicitly defined as a function of time:

(1) $$\begin{array}{c} \psi \left(t\right)=\frac{1}{\sqrt{b\pi }}e^{-{\left(t/b\right)}^{2}}e^{j2\pi f_{c}t}, \end{array}$$

where $j=\sqrt{-1}$, *f*_*c*_ corresponds to the center frequency of the mother wavelet, and *b* is a bandwidth parameter related to the energy spread in the frequency domain.

This mother wavelet will be progressively compressed/stretched in a process called “scalling,” providing a way to decompose the signal in multiple time and frequency scales.

The EMG parameters from time-frequency domain were obtained from the scalogram (image) processing, after applying the wavelet transform through the **Morlet** family and using a scale array (1/*f*) defined from a set of pseudofrequencies (in Hz) related with the typical EMG informational content (0–500 Hz), i.e, 1/*f*=[1/499, 1/497, 1/495,…, 1/9, 1/7, 1/5]=[1.628, 1.635, 1.641,…, 90.278, 116.071, 162.5], as described by Graham et al. [20].

According to this approach, *Major Time* and *Major Frequency* define the coordinates of the centroid:

(2) $$\begin{array}{c} \text{Major Time}=\frac{\sum_{f=1}^{F}\sum_{t=1}^{T}t\times S\left(t,f\right)}{T\times F}, \end{array}$$

(3) $$\begin{array}{c} \text{Major Frequency}=\frac{\sum_{f=1}^{F}\sum_{t=1}^{T}f\times S\left(t,f\right)}{T\times F}, \end{array}$$

where *S*(*t*, *f*) defines the scalogram (2D coordinate system with a third virtual coordinate established by the pixel values/colors) pixel value at coordinates (*t*, *f*), *t* the time coordinate of the pixel, *f* is linked with the frequency dimension, and *T* and *F* are, respectively, the number of available columns and rows of the scalogram (dependent on the chosen frequency and temporal resolutions of the wavelet decomposition Δ*t*=1/*F*_*s*_=1/1000=0.001 *s* and Δ*f*=2 Hz, where *F_s_* is the data acquisition sampling rate).

**Figure 1:**
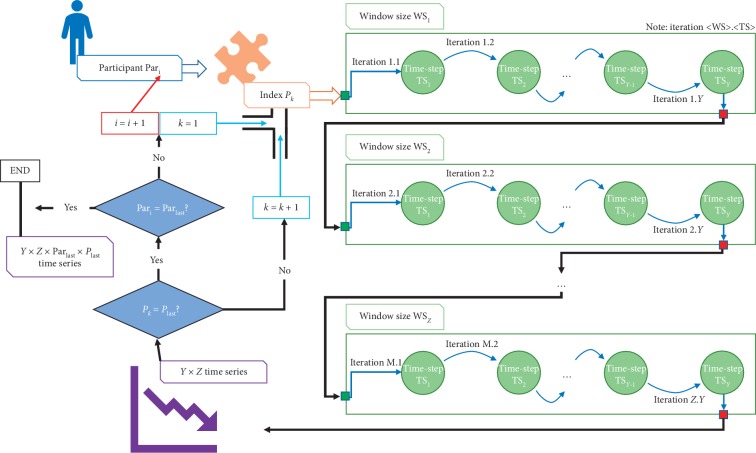
Illustration of the iterative process followed during data processing, so that for each participant all parameters were extracted using the various combinations of window sizes and time-steps (Par_*i*_ | Participant *i*; Par_last_ | Last participant; *P*_*k*_ | Index/parameter *k*; *P*_last_ | Last index/parameter; WS | Window size; WS_*z*_ | Last window size; TS | Time − step; TS_*y*_ | Last time − step).

*Mean Power* corresponds to the average power obtained at scalogram entries' values. *Volume*, *Area*, *Time Dispersion*, and *Frequency Dispersion* are all dependent on a scalogram segmentation phase using the Otsu methodology, excluding low-intensity values [35]. For the remaining values, the convex volume and the convex area were then determined using *Time Dispersion* and *Frequency Dispersion* as the parameters that defined the maximum length of the convex area according to the time and frequency dimensions. The processing scheme used is depicted in Figure 1.

This scheme mainly consists in an iterative procedure where, in each iteration, EMG and HRV data from each participant (Par_*i*_) are analyzed in order to extract all individual parameters (*P*_*k*_) contained in the primordial set (Table 1), using different sliding window mechanism configurations, varying the window size (WS_*z*_ | WS^EMG^=[5,10,15,20,25] muscular activation periods and WS^HRV^=[30,40,50,60,70,80,90,100,110,120]*s*) and time-step (TS_*y*_ | TS=[0,10,25,50,75,90]% of WS_*z*_).

Regarding the window sizes of EMG data, a brief explanation should be provided, taking into consideration that each window is composed by a set of muscular activation periods.

However, the muscular activation periods are analyzed individually, i.e., from each muscular activation period the parameter under evaluation is extracted. Then, the generated set of results is averaged, giving rise to a single value, which represents the overall data inside the window.

Each parameter *P*_*k*_ produced a set of *Y* × *Z* time series, where our meta-analysis took place. Taking into consideration the size of the population under study (11 or 14 subjects), the number of EMG (11 indexes on all 5 muscles) and HRV (14 indexes) parameters, and, obviously, the number of window size (*Z*^EMG^=5 and *Z*^HRV^=10) and time-step (*Y*=6) configurations, it means that we were dealing with a big volume of data to be analyzed, i.e., more than 27390 time series.

#### 2.3.2. Analysis Stage Identification of Trends and Potential Fatigue Descriptors

*(1) Identification of Trends*. Using an adaptation of a meta-analysis procedure, by combining the descriptions of Becker and Wu [29] and Borenstein et al. [36], we identified trends in different time series generated in the *Processing Stage* (by the application of the sliding window mechanism).

Trends were derived from the fitting of a linear regression model to the time series of each parameter (providing a slope value and standard error) and converted to two classes, either as increasing or decreasing trajectory patterns.

Despite the fact that some EMG and HRV parameters may not evolve linearly due to changes on our fatigue variable, by using a linear regression model we decrease the number of degrees of freedom in our analysis, since a relatively complex time series is synthesized in a simple regression line.

Subsequently, the slopes of all participants were reduced into a single combined slope. This final slope was extracted to reflect the overall trajectory of change in the parameter *P*_*k*_ under analysis (generic parameters are used on this section in order to present the applied processing methodology) over time synthesizing in a single value the population trend. This was carried out by combining the slope of parameter *P*_*k*_ for each participant (11 participants for HRV and 14 participants for EMG) and computing a weighted average (of 11 slopes for the HRV indexes and of 14 slopes for the EMG parameters). The weights were computed as the inverse of the square of the respective standard errors (equation (6)). For each participant Par_*i*_, the evolution time series of the index *P*_*k*_ has been generated through a sliding-window mechanism (with the window size WS_*z*_ and time-step TS_*y*_). For the generated time series, the linear regression model was fitted, returning a slope *m*_*i*_ and a variance *σ*_*m*_*i*__^2^. These individual slopes and variances (1 per participant) are joined in a combined slope (weighted average described in equation (4)) that reflects the global behavior of *P*_*k*_ evolution for our population sample.

This method was applied to each one of the previously specified parameters shown in Table 1 and combinations of window sizes (WS_*z*_) and time-steps (TS_*y*_), presented in Section 2.3.1.

(4) $$\begin{array}{c} m_{\text{comb}}=\frac{\sum_{i=1}^{\text{NS}}m_{i}w_{i}}{\sum_{i=1}^{\text{NS}}w_{i}}, \end{array}$$

(5) $$\begin{array}{c} \sigma_{\text{comb}}^{2}=\frac{1}{\sum_{i=1}^{\text{NS}}w_{i}}, \end{array}$$

where NS is the number of participants/subjects (NS^HRV^=11 and NS^EMG^=14) and *w*_*i*_ is

(6) $$\begin{array}{c} w_{i}=\frac{1}{\sigma_{m_{i}}^{2}}. \end{array}$$

Using combined slope (4) and variance (5), we then calculated the associated 95% confidence interval as follows:

(7) $$\begin{array}{c} IC_{\left(1-\alpha \right)\times 100\%}^{m_{\text{comb}}}=\left[{\hat{m}}_{\text{comb}}-F_{t_{\left(n-2\right)}}^{-1}\left(1-\frac{\alpha }{2}\right)\sqrt{{\hat{\sigma }}_{m_{\text{comb}}}^{2}};{\hat{m}}_{\text{comb}}+F_{t_{\left(n-2\right)}}^{-1}\left(1-\frac{\alpha }{2}\right)\sqrt{{\hat{\sigma }}_{m_{\text{comb}}}^{2}}\right], \end{array}$$

where ${\hat{m}}_{\text{comb}}$ and ${\hat{\sigma }}_{m_{\text{comb}}}^{2}$ are the estimated values of the combined slope and variance, respectively; *F*_*t*_(*n* − 2)__^−1^ corresponds to the value of the *t*-test statistic for *n* − 2 degrees of freedom; and *n* represents sample size. The combined slope and the confidence interval were also analyzed graphically using the *Forest Plots* [37].

The computation of the combined slope and variance associated with *P*_*k*_ was repeated for various windows of different sizes and time-step pairs (WS_*z*_ and TS_*y*_, respectively). Then, to assess the quality of each combination, we calculated the coefficient of variation (CV) of WS_*z*_ and TS_*y*_, using the combined standard deviation (*σ*_comb_) and slope (*m*_comb_):

(8) $$\begin{array}{c} \text{CV}\left[{\text{WS}}_{z},{\text{TS}}_{y}\right]=\frac{\sigma_{\text{comb}}}{m_{\text{comb}}}. \end{array}$$

The rationale behind the choice of CV as a statistical indicator of the quality of window-size and time-step combination is related with the fact that well-defined trends should be characterized by (1) representative variations (monitored by the slope) and (2) also by a small uncertainty linked to the regression model fitting stage (monitored by standard deviation).

*(2) Potential Fatigue Descriptors*. As detailed in Section 2.2.2, the experimental protocol also included an acquisition of the heart rate data during recovery from submaximal cycloergometry performed above the VT_2_. The stage of data collection occurring during cycloergometry was termed “acquisition +” and that occurring during postexercise recovery was termed “acquisition −.” These data were used to identify all the parameters that were sensitive to variations in the magnitude of fatigue.

As described on some research works, while the *vastus medialis* muscle exhibits a high level of fatigue-resistance to cycling exercise, this is not the case for the *rectus femoris* muscle [38, 39]. Thus, for the EMG parameters, “acquisition +” and “acquisition −” data were obtained in the *rectus femoris* and *vastus medialis*, respectively (two antagonistic conditions, where fatigue patterns will be more evident or undefined/nonexistent, respectively).

On the current stage of the project we focused our analysis on 2 of our 5 muscles' protocols, the choice of 5 muscles is intended to expand, in the near future, the analysis of the pilot study; however, the data processed for the remaining 3 muscles were extremely useful to confirm the theoretical expectations collected from other research articles that fatigue-induced patterns on EMG parameters are more accentuated on the *rectus femoris* and less prominent on the *vastus medialis* muscle [38–40].

In scientific terms, there is a general agreement that the study of a single case/participant or, in our case, a single trend is not enough to draw definitive physiological conclusions [41]. To overcome this problem, we established two criteria to distinguish between generic- and fatigue-associated trends for each one of the EMG and HRV indexes under analysis.

*Premise*: For each parameter *P*_*k*_, the trend evaluation procedure will be restricted to the most representative combination of (WS_*z*_ and TS_*y*_) that describes the evolution of *P*_*k*_ (i.e., the combination that minimized the CV).

**Figure 2:**
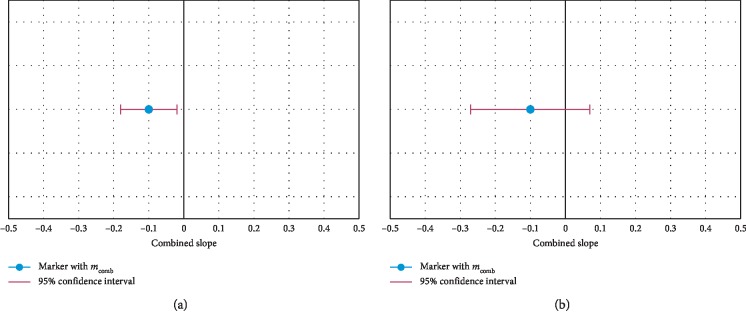
*Forest Plots* graphically synthesizing the results of the meta-analysis, based on the estimate of the combined slope and the respective confidence interval. (a) Graphical result of the meta analysis when a trend exists. (b) Graphical result of the meta analysis when no trend exists.

 
*Trend Acceptability Criteria*:
(1) Taking into consideration the previous premise, for the “acquisition +”, it is considered the existence of a trend only if the confidence interval, linked to the combined slope estimate of parameter *P*_*k*_, did not cross the center of the *Forest Plot* (zero slope), belonging exclusively to one of its two domains (Figure 2(a))
(2) The trend identified on “acquisition +” is only considered a fatigue-related trend when it was verified in the “acquisition −”, a combined slope reversal in *P*_*k*_ vs. that seen in “acquisition +”, or at least a state of uncertainty, where confidence intervals included values of the two domains of the *Forest Plot* (Figure 2(b))

These criteria were applied sequentially. We established that *P*_*k*_ descriptor did not correspond to a fatigue indicator whenever one of these criteria was not fulfilled.

Therefore, a parameter *P*_*k*_ is considered an individual fatigue descriptor (IFD) if the provided algorithm steps (section A of Supplementary Material ([Supplementary-material supplementary-material-1])) are followed.

### 2.4. Proposed Solutions: Global Fatigue Descriptor and Binary Classifier

Finally, the *Proposed Solutions* (third stage of the research process, defined on Section 2.3) included the definition of a global fatigue descriptor (GFD) and implementation of a binary classification system. At this point, all the information provided by the EMG and HRV indexes was modeled into a single value that characterized the GFD. Then, we generated a classifier with the ability to process the information of the *Processing Stage*, i.e., the selected features derived from the identified IFD as an input and returning an output containing one of the two classes (+ fatigue vs. − fatigue).

The GFD corresponds to a weighted average, where each of the individual fatigue descriptors was normalized. It defines an input with a weight equivalent to the inverse of the CV, determined at the end of the application of the trend identification methodology (equation (9)).

GFD is a dimensionless parameter that may present a value between 0 and 1:

(9) $$\begin{array}{c} \text{GFD}\left[i\right]=\frac{\sum_{k=1}^{K}{\text{IFD}}_{k}\left[i\right]\times {\text{CV}}_{k}^{-1}}{\sum_{k=1}^{K}{\text{CV}}_{k}^{-1}}, \end{array}$$

where K is the number of fatigue descriptors to be considered, IFD_*k*_[*i*] being a sample of the fatigue descriptor k, and CV_*k*_ being the CV for the most favorable window-size and time-step combination for the extraction of the IFD_*k*_ descriptor.

**Figure 3:**
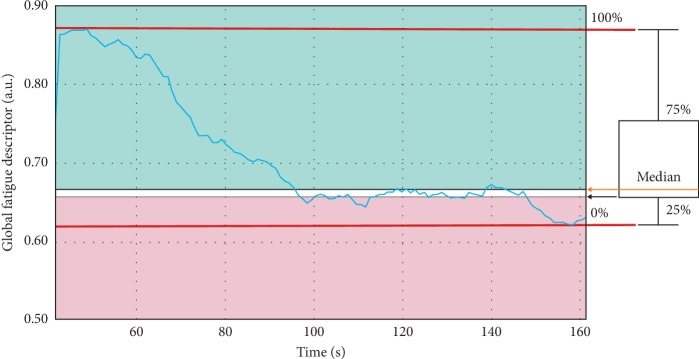
Graphical correspondence between the thresholds defining the fatigue zones and their origin in the *box plot*.

A calibration protocol, similar to the one described in Section 2.2.2, was applied to each participant's data. This allowed representing the evolution of GFD over time in a graphical figure. The calibration provided the maximum values of each IFD used for normalization. Afterwards, its distribution was computed and graphically translated into a *boxplot* (Figure 3). Consequently, a profile of each participant became available to the system based on the segmentation of the GFD into three regions (green, yellow, and red), using two thresholds corresponding to the 25% percentile and the median value. These regions were set to assess the magnitude of fatigue.

The proposal of 50% (median) and 25% as GFD thresholds was mainly based on statistical concepts.

In first place, the 50% threshold was defined, taking into consideration the intention of segmenting the GFD values into two equally probable regions (for the known population being the median value, less susceptible to outliers when compared with the mean), i.e., the green and red zones, representative of innocuous and dangerous fatigue levels, respectively.

**Figure 4:**
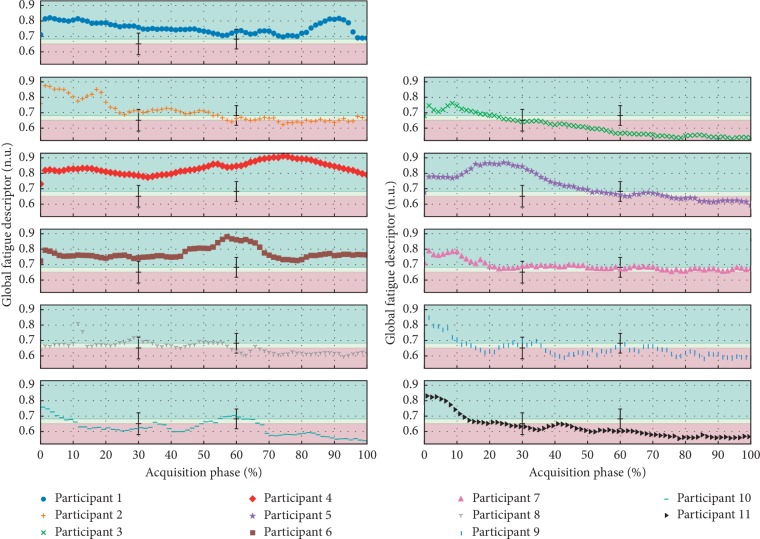
Presentation of the evolution of the GFD for the various participants that compose the population sample (“acquisition +”), highlighting the regions of separation between fatigue levels.

Nevertheless, after analysing the first results (Figure 4) it was concludedthat, in a great majority of the cases, the GFD evolution is characterized by a first stage of abrupt decline followed by a prolonged interval of stability, something that could be related with the fatigue acquisition mechanism specificities.

In fact, the physiological process of fatigue acquisition can also be divided into two stages: (1) aerobic and (2) anaerobic, accordingly to the dominant mechanism of cellular energy production.

In the beginning of the exercise, the dominant energy production mechanism is the aerobic respiration, being considerably more efficient, but also slower than the anaerobic type.

After reaching the maximum rate of aerobic energy production, the fastest anaerobic respiration gradually starts becoming dominant, in order to suppress the energetic needs.

The beginning point of this aerobic-anaerobic transition period is defined as the first lactate threshold, which may be linked with the start of the identified “stability” period in the GFD evolution [42, 43].

However, in this transitional period, it is neither a severe/dangerous (red) or innocuous (green) state of fatigue, creating the need of including an additional category, i.e., the yellow zone.

Replicating the splitting logic applied to green and red regions, the original red region was divided into two equally probable intervals, through the 25%, giving rise to the final scale composed of three fatigue levels.

With regard to the binary support vector machine fatigue classifier (the creation and training were implemented based on Python Scikit-learn, a library specialized in machine learning), the two implemented classes corresponded to “nonfatigued” and “fatigued,” identifying when each participant was in its optimal physical condition or under fatigued conditions, respectively.

The SVM model is very attractive and intuitive, taking into consideration that it essentially works based on a Cartesian logic, where each example is a point (contained inside the Cartesian space) univocally defined by *N* coordinates, i.e., each feature corresponds to a dimension of the space.

This supervised machine-learning model should be included in the hyperplanes' class, where, through a finite but representative set of training examples, the major aim is to find a function *f* (decision function) capable of properly returning an output/prediction (class) after receiving a new testing example as input [44].

In order to achieve this end (identification of the decision function *f*), the training phase of a SVM is based on the search for the hyperplane ($\vec{w}\cdot \vec{x}+b=0,\vec{w}\in R^{N},b\in R,$$\vec{x}$ being the vector with the value of the features associated with the testing example and $\vec{w}$ and *b* being the parameters determined during the classifier training phase) that ensures the maximization of the separation between the two groups of training examples, which are geometrically defined by two convex hulls.

With the two groups of training examples well delimited, the next stage consists in an optimization procedure intended to identify the smallest segment that links the boundaries of the two convex hulls. The optimum hyperplane is perpendicular to this segment and crosses its central point.

The previous logic will produce the pretended decision function *f*:

(10) $$\begin{array}{c} f\left(\vec{x}\right)=\text{sign}\left(\vec{w}.\vec{x}+b\right),\vec{w}\in R^{N},b\in R. \end{array}$$

This type of classifier was initially intended for binary problems [45], which fully meet our needs to categorize fatigue state into two classes.

Our aim is not focused on defining an absolute threshold of fatigue, but instead a qualitative measure of when fatigue is being acquired or not and the respective relative level of installation.

Returning to the specific implementation of the binary classifier, the procedure for preparing the set of training examples was based on the segmentation of EMG and HRV signals, from the 11 available participants into two temporal segments with equal duration. From the first segment, the features of the “nonfatigued” class examples were extracted, while the other half segment (related to the final period of the trial) was used to extract the information for the training examples of the “fatigued” class.

Two features were extracted from each time series for the *N*+*M* fatigue descriptors (*N*=4 and *M*=10 being the number of EMG and HRV fatigue descriptors, respectively). This was carried out to include information about their absolute values and trends in the training phase. The formulation of these features corresponds to the average value of the normalized series (equation (11)) and the relative variation rate (equation (12)):

(11) $$\begin{array}{c} {\text{feature}}_{1}=\frac{{\sum^{}}_{j=1}^{L}P_{k}\left[j\right]/{\text{max}}_{P_{k}}}{L}, \end{array}$$

(12) $$\begin{array}{c} {\text{feature}}_{2}=\frac{\left(P_{k}^{\text{end}}/P_{k}^{\text{start}}\right)-1}{t_{\text{end}}-t_{\text{start}}}, \end{array}$$

where *L* represents the number of samples of the parameter *P*_*k*_ extracted from the analyzed segment of the EMG or RR_tachogram_ and max_*P*_*k*__ represents the maximum value of the *P*_*k*_ descriptor for normalization. The terms of feature_2_, *P*_*k*_^start^, and *P*_*k*_^end^ correspond to the *P*_*k*_ parameter in the first and last sample of the time series and *t*_start_ and *t*_end_ to the respective time instants.

**Figure 5:**
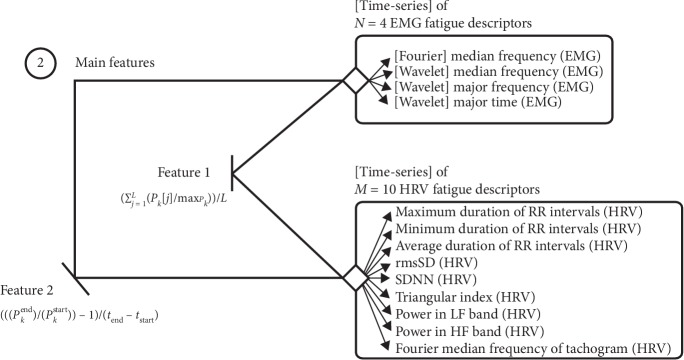
Diagram presenting the initial set of 28 features (before feature selection), obtained through the extraction of 2 parameters (feature_1_ and feature_2_) from the time series of *N*=4 EMG and *M*=10 HRV fatigue descriptors (described in Section 2.3.1 and identified on Section 3).

The training phase was preceded by the selection of features using recursive feature elimination [46]. Before feature selection, each one of the 22 training examples (11 for the “nonfatigued” class and 11 for the “fatigued” class) was characterized by an array with 2 × (*N*+*M*)=28 entries (Figure 5).

The classifier provides an assigned class together with an estimate of its degree of certainty (i.e., a qualitative and quantitative output).

## 3. Results

Using the methods described in Section 2.3.2, we obtained a list of potential fatigue descriptors (that we previously refer as IFD) consisting of 4 EMG and 10 HRV parameters, as summarized in Table 2.

Table 2 shows the typical evolution trend, the combination of window size, and time-step that provides a more efficient analysis of the descriptor and the respective CV.

Due to the GFD definition, as almost all of its inputs (IFD) decrease with the onset of fatigue, this is the typical behavior for the GFD in most participants. Figure 4 emphasizes the observed evolution for the 11 participants, presenting valid EMG and HRV acquisitions, while Table 2 defines the identified weights contained inside the formal definition of GFD. The threshold values, between colored zones, were determined by averaging the thresholds obtained for each participant on the calibration protocol. The error bars illustrate the standard deviation associated with the calculated mean value. As for the classification, the results highlight how class and GFD evolve in parallel for most of the participants.

Regarding the feature selection stage, the original training array was composed by 28 entries. The feature selection procedure generated a considerable reduction in the number of meaningful features, which decreased to 9.

We executed a recursive feature elimination stage through a Leave One Out cross-validation strategy. With this strategy, different sets of features were tested sequentially, removing in each iteration the less meaningful feature until reaching the limit case, where only one feature remained. The performance of the trained classifiers (1/iteration of the cross-validation procedure) was evaluated. The complete list of classifiers' score provided a way to understand which is the most effective set of features to train the binary classifier.

The feature_1_ was considered the most relevant for a set of 9 parameters correspondent to **HRV Group** | *Average RR*, *SDNN*, *Triangular Index*, *SD2*, *Fourier Median Frequency*, *Power in LF Band* and *Power in HF Band*, **EMG Group** | *Wavelet Median Frequency* and *Wavelet Major Frequency*.

**Figure 6:**
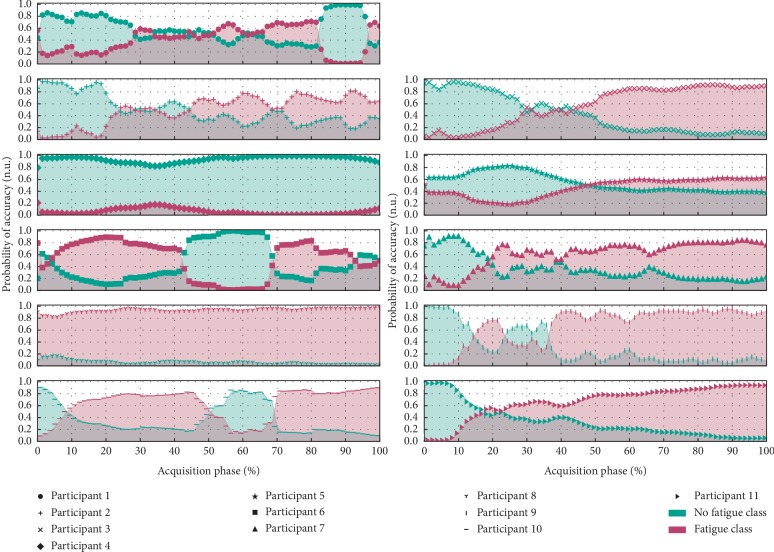
Demonstration of the evolution in the classification attributed by the support vector machine throughout the “acquisition +” collected in the various elements of the population sample.

Regarding the results of the trained classifier, we found that, in the initial phase of the test, the “nonfatigued” class was assigned with a high degree of certainty. Then, the certainty of assignment of this class decreased gradually until the “fatigued” class began to be dominant towards the end of the exercise trial (Figure 6). Feature extraction in the test examples differs slightly from the training phase procedure, disregarding averages and using instantaneous values so that classes can be returned over time, during the real-time processing of data.

For evaluating the quality of the classifier, a cross-validation method based on a *Stratified K-Fold* (*K* = 11 folds) strategy was applied, providing an accuracy score of 0.82 ± 0.24 with a 95% confidence interval between [0.68; 0.96].

## 4. Discussion

In this study, we implemented a monitoring system capable of showing trends on the evolution of EMG and HRV parameters during cycloergometry, using an indirect and noninvasive approach to qualitatively evaluate the fatigue progression.

One of our main concerns was related with the applicability of EMG parameters extracted from the Fourier domain, taking into account that we were dealing with dynamic muscular contractions, where the stationarity criterium can be affected due to changes in force, speed, muscle length, and electrode relative location along the cycling trial [47–49].

In order to avoid a possible problem of nonstationarity EMG, wavelet analysis was conducted, considering that it is not based on the assumption of stationarity [50–52].

However, we are still interested on exploring methodologies that require less computational resources, such as short-time Fourier transform (STFT), which is based on the conventional Fourier transform but applied on a sequence of short sliding windows. Direct application of the Fourier transform in EMG data collected during dynamic/cyclic tasks is not appropriate [48], but STFT will be viable if the size of the time windows under analysis is sufficiently short to avoid the effect of nonstationarities during the application of the Fourier transform [47, 49, 50, 52–54]. The common size of our EMG processing windows is approximately equal to 1 second, corresponding to the muscular activation period, which is in accordance with some studies executed in similar experimental conditions [49, 52].

Before proceeding with the discussion, it should be noted that, in our analysis, each window is composed by multiple muscular activation periods. As an illustrative example, the most favorable window-size configuration to extract *Fourier Median Frequency* (Table 2) will be composed by a set of 10 muscular activation periods.

Each muscular activation period is a subwindow, and from each of these subwindows, the respective Fourier power spectrum is generated and the *Fourier Median Frequency* is determined.

So, returning to our illustrative example, each window originally generates 10 *Fourier Median Frequency* values, which will be averaged, giving rise to an overall *Fourier Median Frequency* value for the window under analysis.

With this approach, in each window, it is ensured that (1) the stationarity conditions are fulfilled, taking into account that each power spectrum was generated from subwindows (individual muscular activation's) with less than 1 second and (2) the average of the set of *Fourier Median Frequency* values minimizes the influence of outliers.

Regarding HRV analysis, in spite of the fact that a relationship between physical fatigue and HRV patterns can be established [55–56], the most common applications are related with subjective/mental fatigue [57–59].

As declared before, the projection of our computational system with both EMG and HRV parameters was intended to collect patterns related with physical and subjective fatigue, ensuring a global evaluation of the fatigue state.

So, with HRV parameters, we believe that it is possible to reach subjective/mental fatigue patterns and also complement the physical/local information gathered with EMG.

Taking these concerns into consideration, several EMG and HRV parameters were modeled with the purpose of identifying trends described by the global fatigue index that was extracted from the data obtained during and after exercise performed above the VT_2_.

The trend identification phase gave rise to an extensive list of fatigue descriptors.

Identified trends were reasonably supported by past study results, namely, the decrease of EMG median frequency [3, 5], wavelet parameters [20], SD2, absolute power in LF and HF bands [56, 60, 61] and increase of heart rate (inverse of RR interval duration) [60].

Then, the descriptor list was refined, through a pretraining feature selection methodology, to avoid redundancies in the definition of the global fatigue descriptor and in the training of the support vector machine (Table 2). As referred previously and taking into account that the global fatigue descriptor is defined as an average of these individual fatigue descriptors, it denotes a decreasing trend with the progression of the trial, evolving in parallel with the probability of accuracy in the decision returned by the binary classifier.

These results (trend identification, evolution of the global fatigue descriptor, and training of the binary classifier) show different dependencies with regard to the coefficient of variation. The coefficient of variation enabled detecting the best sliding window mechanism combination (window size and time-step) for generating the evolution time series of each EMG and HRV index contained in the preliminary list (Table 1). This is fundamental for all the subsequent research, since it is from these time series that trends were identified and individual fatigue indexes were found and used next at the definition of the global fatigue descriptor and during the training of the classifier. Among the different assessment possibilities and considering the combined slopes and standard deviations here obtained, our approach emerged as a reasonable method to optimize the sliding window mechanism procedure for extracting each descriptor. Regarding the trained classifier, its main purpose is to distinguish data into two extreme conditions: Fatigued and Nonfatigued, but we were also concerned in tracking how the system behaves in the intermediate state between these two extreme conditions, which, as stated in Section 2.4, can be achieved through a quantitative metric: Degree of Certainty.

So, the classifier has two outputs: **(**1) a qualitative result (class: [Fatigued, Nonfatigued]) and (2) a quantitative result (degree of certainty).

If the main focus was only the qualitative result, the chosen training approach (splitting the training data into two half-size temporal segments) could create some problems, because, in practical terms, it will be possible that data collected in the beginning of the trial and in the middle will produce the same qualitative result (Nonfatigued class). But, if both qualitative and quantitative outputs (returned by the classifier) are taken into account, the previous undesirable situation can be avoided and the system is able to distinguish data collected from the beginning and middle of the trial, because the degree of certainty in the middle will be considerably lower than in the beginning (as demonstrated by almost the full set of results available on Figure 6).

In spite of the binary nature of the classifier, when it returns the Nonfatigued class as a result of input test data, the system is not stating in an absolute way that the test data are related to a Nonfatigued state, giving, instead, a probability of this state.

Of course, to guarantee the reliability of the used approach (for segmenting the training data), some assumptions were needed:

1. At the beginning of the trial, the volunteer should be in an absolute Nonfatigued state, which was guaranteed by the resting hours and absence of intense physical activity on the hours precedent to the trial
2. The variable under study (“Fatigue”) is incremented during the trial, which means that the volunteer state is gradually becoming away from the Nonfatigued class and entering in the Fatigued one
3. At the end of the trial, it is assumed that the volunteer was in an absolute Fatigued state, which is a reasonable consideration taking into account the exhaustion and inability to proceed when the exercise was reached
4. Fatigue is acquired in a constant rate, condition reasonably ensured by the constant work rate used on the cycloergometry test

**Figure 7:**
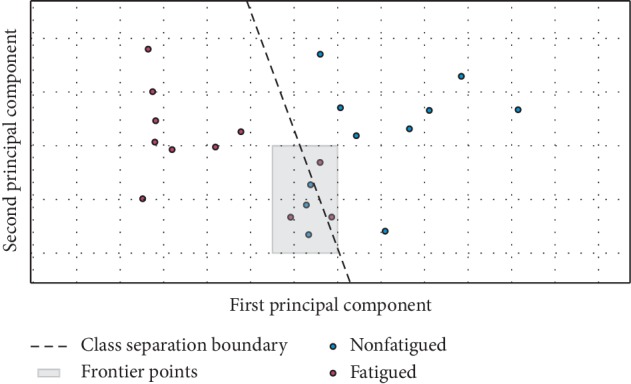
Demonstration of viability of reducing the dimesionality of the classification system, taking into consideration that with only 2 principal components a very reasonable separation between “Fatigued” and “Nonfatigued” class is ensured.

In terms of the feature selection stage of the SVM classifier, it should be refined in the future (removing some redundant components), taking into consideration that after performing a Principal Component Analysis (PCA), it was concluded that 79% of the informational content intrinsic to the 9 features can be ensured by using only 2 principal components, i.e., the dimensionality of our classifier can be considerably reduced (as demonstrated by PCA and by the produced graphical results presented in Figure 7).

In fact, reducing the dimensionality of the classifier produced very interesting results in the performance evaluation stage through a *Stratified k-Fold Strategy*. The average classification accuracy decreased a little (from 82% to 77%), but the 95% confidence interval has shrunk from ±24% to ±19%, which means that the classification system was simplified but kept its effectiveness.

After a careful analysis of the original results, it was noticed that the classification accuracy could be further improved.

This increase of accuracy was achievable with a simple adjustment in the training stage of the classifier. Instead of splitting the acquired EMG and HRV signals into two halves (the first half, representative of the “nonfatigued” class, while the second is linked to the “fatigued” class), training data from the “nonfatigued” class can be extracted from the 1^st^ quarter and the “fatigued” data from the 4^th^ quarter segment of the original full-time acquisitions.

With the previous approach, for each class, an increase on the specificity of the training examples is ensured, taking into consideration that the excluded EMG and HRV data (from the 2^nd^ and 3^rd^ quarter segments of each trial) are related to a transition stage between “nonfatigued” and “fatigued” classes and not exclusively to one of these two classes.

**Figure 8:**
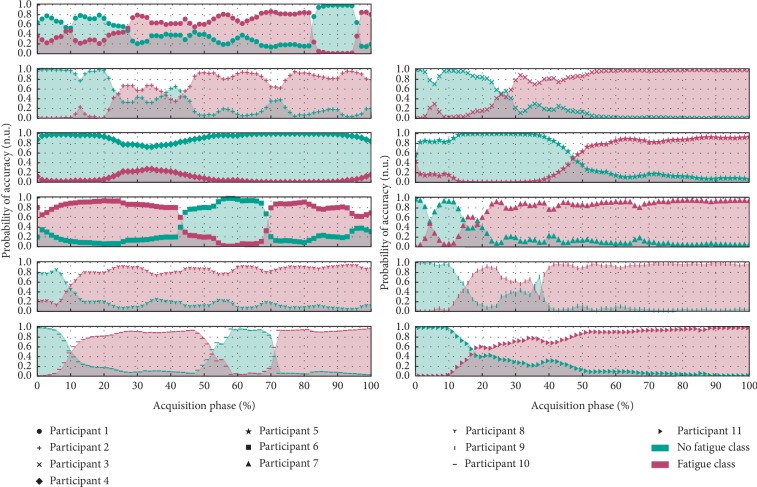
Evolution in the classification attributed by the support vector machine throughout the “acquisition +” collected in the various elements of the population sample after reviewing the segmentation methodology of the training data.

Through the previous adjustments in the feature selection/training stage, the estimated overall classification accuracy increased to 95% ± 8%, while the practical results, using a sliding-window mechanism to evaluate the evolution of fatigue instantaneous classification, were maintained (as demonstrated in Figures 6 and 8).

The combination of information from different fatigue descriptors relied on the GFD and support vector machine. Nevertheless, it should be considered that the GFD corresponds to an exploratory approach that still needs a more profound validation. For instance, the definition of GFD thresholds is based on a statistical criterion, while deeper physiological correspondences could be relevant, i.e., the present results are promising, although a strong physiological connection/meaning should be found, in order to achieve a more solid interpretation.

### 4.1. Limitations

Currently, this classification system is tuned for acquisitions similar to those reported in the experimental protocol (during a cycling task with the monitoring of *rectus femoris* muscle). In a first look, it could be expected that this restrictive training procedure would entail an undesired biased behavior of the classifier (with more false positives). We believe that the choice of using only one muscle at the training phase will not cause a biased behavior, which would be problematic, because we have two well-defined acquisition segments representative of “nonfatigued” and “fatigued” classes. However, it is probable that the classifier reveals an excess of specificity (working well for data acquired from *rectus femoris* and worst for other muscles).

When tested with other muscles, or motor tasks, the classifier accuracy might be compromised because the manifestation of fatigue varies substantially between different muscles and exercise paradigms [62]. In line with this concept, new acquisitions will be extremely important to further overcome these limitations and allow a more generalized and accurate tool for monitoring the onset of fatigue noninvasively.

The present version of the system also requires a calibration test for a correct operation, which is essential for IFD normalization. Normalized values, used as inputs of GFD and for generation of the support vector machine training arrays, decrease the impact of the variability in the fatigue acquisition process, typical between participants.

Additionally, the dimension of the population sample should be increased in order to achieve more solid generalizations and to reduce unexpected behavior of the binary classifier and global fatigue descriptor. This need is reinforced by the results reported for participants 1, 6, and 10, where the binary classifier suddenly flags an abrupt recovering period in the middle/end of the trial.

As previously referred, physiological patterns of fatigue are considerably subject specific, which means that by increasing the training population, the resultant model will ensure a better generalization.

However, the size of the population sample is probably not the only reason for the reported short-term (participants 1, 6, and 10) and long-term (participants 4 and 8) behavior. The trained SVM model was based on a linear kernel due to its computational elegance and simplicity [44].

In spite of requiring less computational resources, its simplicity may create some rigidity on the definition of the hyperplane responsible for separating the two classes under analysis (“nonfatigued” vs “fatigued”), taking into account that geometrically it corresponds to a straight line in a 2D space.

For increasing the generalization/adaptability of the model, in the future, the kernel could be changed to a polynomial or a radial basis function (RBF) type, which could achieve a better separation of frontier points (as the ones highlighted on Figure 7), decreasing the occurrence of incorrect classification results.

## 5. Practical Applications

The described functionalities were implemented as a plugin of *OpenSignals* software, which emerges as an intuitive tool for processing physiological signals acquired by the systems designed and marketed by PLUX Wireless Biosignals, namely, in the *biosignalsplux* (PLUX Wireless Biosignals, 2015). The interface provides some sections for user interaction intended to the configuration of the processing algorithms, presenting the results divided into four zones, including EMG and HRV events detected (periods of muscle activation and *R* peaks) and the evolution of each of the IFD, the GFD, and the support vector machine class assigned.

With these processing functionalities, the computational system defines an interesting solution that can be applied in research studies and even by coaches and athletes, helping to prevent overtraining condition and ultimately the occurrence of muscular injuries.

## 6. Conclusion

In the final analysis, we can conclude that 4 EMG (*Median Frequency*, from Fourier and Wavelet analysis; *Major Frequency*; *Major Time*) and 10 HRV parameters (*Maximum*, *Minimum* and *Average RR Interval*; *SDNN*; *rmsSD*; *Triangular Index*; *SD2*; *Power in LF* and *HF Band*; *Median Frequency*), i.e., 14 individual fatigue descriptors, exhibited a tendentious behavior over time in the participants included in our population. This tendentious behavior, according to the proposed trend evaluation methodology, is a demonstration of the correlation between the variable under study (fatigue) and the selected EMG and HRV parameters.

Taking into consideration the previously stated importance of the coefficient of variation to qualify the degree of correlation between changes in the experimental variable and the evolution of the extracted parameters, it can be concluded that *Fourier Median Frequency* will be the best EMG fatigue descriptor while *Average and Minimum RR Intervals* duration are the most meaningful HRV parameters, due to the lower CV values.

Combined information from these descriptors was achieved by the definition of our own global fatigue descriptor, an index that reflects the simultaneous impact of fatigue at the neuromuscular and cardiac autonomic level (objective nature) and also in a more global perspective when including HRV parameters, relevant indicators of mental/subjective fatigue. Since the implemented processing system extracts all information using a sliding window mechanism, a future adaptation of the system to a real-time analysis can be facilitated. The actual version is interesting within the context of exercise training because it may aid the estimation of the optimal individual workload during an acute exercise session. Ultimately, within the context of chronic endurance exercise, we speculate that this approach might be valuable for prophylaxis against overtraining and its negative side effects [63, 64].

A future implementation could benefit from the previously mentioned real-time algorithm. This would provide the user an automatic and immediate *feedback* about fatigue development.

In the work presented, our promising results on combined fatigue indices and the related classification approach are already providing novel tools for fatigue assessment that pave the way for broader, more robust, studies with larger populations that could potentially be established as preventive fatigue assessment mechanisms.

## Figures and Tables

**Table 1:** List of the various EMG and HRV parameters studied.

EMG			ECG/HRV	
Time domain	Frequency domain	Time-frequency domain	Time domain	Frequency domain
RMS^†^	Median frequency	Median frequency	Maximum RR interval	Power inside ULF band^∠^
	Total power	Major frequency	Minimum RR interval	Power inside VLF band^∡^
		Major time	Average RR interval	Power inside LF band^∢^
		Mean power	SDNN^‡^	Power inside HF band^*⋇*^
		Area	rmsSD^*∗*^	Median frequency
		Volume	Triangular index	
		Time dispersion	SD1^*⋆*^	
		Frequency dispersion	SD2^*⋆*^	
			SD1/SD2	

^†^RMS, root mean square; ^‡^SD, standard deviation of NN intervals; ^*∗*^rmsSD, root mean square of successive differences; ^*⋆*^Poincaré Standard Deviation/Dispersion of points perpendicular (SD1) or along (SD2) the axis of line-of-identity (ellipse semiaxes 1 and 2). ^∠^ULF, ultralow frequency band ([0; 0.003] Hz); ^∡^very low-frequency band ([0.003; 0.040] Hz); ^∢^low-frequency band ([0.040; 0.150] Hz); ^*⋇*^high-frequency band ([0.150; 0.400] Hz).

**Table 2:** List of the various EMG and HRV fatigue descriptors with the typical evolution trend in fatigue conditions and the most appropriate combination of window size and time-step.

Signal	Domain	Parameter	Window size	Time-step	Coefficient of variation	*m*_comb_±Half length of 95% CI^♠^	Typical evolution
EMG	Frequency	Median frequency	10 muscular activations	1 muscular activation	1.22 × 10^−2^	−3.01 × 10^−2^ ± 8.02 × 10^−4^	↓
EMG	Time frequency	Median frequency	10 muscular activations	1 muscular activation	2.42 × 10^−2^	−2.87 × 10^−2^ ± 1.52 × 10^−3^	↓
EMG	Time frequency	Major frequency	10 muscular activations	1 muscular activation	1.70 × 10^−2^	−3.39 × 10^−2^ ± 1.26 × 10^−3^	↓
EMG	Time frequency	Major time	25 muscular activations	0 muscular activations	3.94 × 10^−2^	−5.19 × 10^−4^ ± 4.46 × 10^−5^	↓
HRV	Time	Maximum duration of RR intervals	30 s	3 s	1.25 × 10^−2^	−1.82 × 10^−1^ ± 5.16 × 10^−3^	↓
HRV	Time	Minimum duration of RR intervals	30 s	3 s	8.94 × 10^−3^	−1.71 × 10^−1^ ± 3.46 × 10^−3^	↓
HRV	Time	Average duration of RR intervals	30 s	3 s	9.13 × 10^−3^	−1.76 × 10^−1^ ± 3.64 × 10^−3^	↓
HRV	Time	rmsSD^*∗*^	50 s	45 s	1.92 × 10^−2^	−3.87 × 10^−3^ ± 1.68 × 10^−4^	↓
HRV	Time	SDNN^‡^	30 s	3 s	5.17 × 10^−2^	−6.09 × 10^−3^ ± 7.12 × 10^−4^	↓
HRV	Time	Triangular index	60 s	30 s	2.04 × 10^−2^	−4.01 × 10^−2^ ± 1.85 × 10^−3^	↓
HRV	Time	SD2^*⋆*^	30 s	3 s	4.25 × 10^−2^	−1.09 × 10^−2^ ± 1.05 × 10^−3^	↓
HRV	Frequency	Power in LF band^∢^	30 s	3 s	5.78 × 10^−2^	−1.51 ± 1.97 × 10^−1^	↓
HRV	Frequency	Power in HF band^*⋇*^	30 s	3 s	5.41 × 10^−2^	−8.51 × 10^−1^ ± 1.04 × 10^−1^	↓
HRV	Frequency	Median frequency	30 s	3 s	1.51 × 10^−2^	3.65 × 10^−3^ ± 1.25 × 10^−4^	↑

^♠^ *m*_comb_ refers to the combined slope and CI to the confidence interval (*m*_comb_±**half length of 95% CI**). ^‡^SDNN refers to standard deviation of NN intervals; ^*∗*^rmsSD refers to root mean square of successive differences; ^*⋆*^Poincaré Standard Deviation/Dispersion of points perpendicular (SD1) or along (SD2) the axis of line-of-identity (ellipse semiaxes 1 and 2). ^∢^Low-frequency band ([0.040; 0.150] Hz); ^*⋇*^high-frequency band ([0.150; 0.400] Hz).

## Data Availability

The acquired physiological data used to support the findings of this study are also restricted, in order to protect patient privacy. However, data may become accessible in some exceptional cases for researchers who meet the criteria for access to confidential data, through the corresponding author Guilherme Ramos (gramos@plux.info).

## Supplementary Materials

Supplementary Materials A. Algorithm for Identification of IFD.
