# Supplementary material for: Fatigue Evaluation through Machine Learning and a Global Fatigue Descriptor
Source: J Healthc Eng. 2020 Jan 6;2020:6484129. doi: 10.1155/2020/6484129 (PMC6969995; doi:10.1155/2020/6484129)
Supplement: Supplementary Materials — A. Algorithm for Identification of IFD. [file 6484129.f1.pdf]

A. Supplementary Material | Algorithm for Identification of IFD

Study Conditions

EMG_{param} = [EMG parameters of Table 1]

HRV_{param} = [HRV parameters of Table 1]

$WS_{EMG} = [5, 10, 15, 20, 25]$ muscular activation periods

$WS_{HRV} = [30, 40, 50, 60, 70, 80, 90, 100, 110, 120]$ s

$\#Participants_{EMG} = 14$

$\#Participants_{HRV} = 11$

Study Conditions Applied to the Algorithm

$\{signal\} = EMG$ **or** HRV

Generic Algorithm

```
foreach P_k in {signal}_param do
    WS_best = None
    TS_best = None
    CV_best = ∞
    foreach WS_z in WS_{signal} do
        TS = [0, 10, 25, 50, 75, 90]% of WS_z
        foreach TS_y in TS do
            m_array = []
            σ²_array = []
            for n = 1 to #Participants_{signal} do
                if CV[WS_z; TS_y] < CV_best then
                    1) Generation of the evolution time series of P_k for a sliding
                       window configuration of window size WS_z and time-step TS_y
                    2) Determination of the regression curve that best fits the time
                       series generated in 1
                    3) Storage of slope m_n inside m_array
                    4) Storage of variance σ²_n inside σ²_array
                end
            end
            1) Determination of m_comb and σ²_comb, accordingly to the mathematical
               formalism defined in equations 4 and 5
            2) Determination of CV[WS_z; TS_y] (equation 8)
            if CV[WS_z; TS_y] < CV_best then
                WS_best = WS_z
                TS_best = TS_y
                CV_best = CV[WS_z; TS_y]
            end
        end
    end
    For the combination [WS_best; TS_best], application of criteria (section 2.3.2) in
      order to verify if P_k is and Individual Fatigue Descriptor (IFD)
end
```
